# Convulsions from Disentery and Intermittent Fever

**Published:** 1846-05

**Authors:** 


### CONVULSIONS FROM DYSENTERY AND INTERMITTENT FEVER.

On the 7th of April, about 4 o'clock in the afternoon, a practitioner of this city was called, in quick succession, to a child two years of age, and a boy aged ten years, both of whom had been seized with convulsions about the same moment. A storm of wind and rain had prevailed during the day. The convulsions in the child were violent, and were followed by dysenteric symptoms which persisted for several days. In the boy the system was less convulsed, and he remained comatose for more than an hour—in fact, until relieved by venesection, which restored him to consciousness. The pupils of his eyes were much dilated, and his pulse was intermittent. During the autumn he had suffered with chills and fever, and they had recurred once or twice in the course of the winter. At this time the attack had been preceded by a cold, with pleuritic symptoms. Calomel and ipecac. were administered, after the venesection; his side was blistered, and on the subsidence of fever quinine was given. Two days after the convulsion a febrile paroxysm occurred, during which the pain in his side and head was much increased, and his cough was almost incessant. By the employment of quinine other paroxysms were obviated, and he had a rapid convalescence.

In the few days of this boy's illness, his father, mother, and grandmother had each a chill. In the fall as many as seven of the family, or about half, sometimes suffered with the chills on the same day. Quinine was found effectual in arresting the disease for the time; but on slight exposure it returned. Iron was the remedy indicated under such circumstances.

